# Supplementary material for: Are carbon emissions trading and green financial instruments synergistic? -Comprehensive quantitative research based on content analysis
Source: PLoS One. 2024 Mar 7;19(3):e0298601. doi: 10.1371/journal.pone.0298601 (PMC10919601; doi:10.1371/journal.pone.0298601)
Supplement: S1 Data — (ZIP) [file pone.0298601.s001.zip › Data/地级市Z_F工作报告.zip!/╜¡╦╒/╤∩╓▌╩╨/└┤╫╘╣1⁄2╓┌║┼í╛┬φ┐╦╩2╛▌═°í┐_2007.docx]

各位代表：

现在，我代表扬州市人民政府，向大会作工作报告，请予审议，并请市政协各位委员和其他列席人员提出意见。

2006年政府工作回顾

2006年是“十一五”发展的开局之年。我们在中共扬州市委的领导下，依靠和带领全市人民，以邓小平理论和“三个代表”重要思想为指导，贯彻落实科学发展观，围绕“两个率先”和“全面达小康，建设新扬州”的目标，解放思想，真抓实干，在跨越发展、科学发展、和谐发展上迈出新步伐，全面完成市五届人大四次会议确定的各项任务，实现了“十一五”时期的良好开局。

1、经济持续快速发展，综合实力明显增强。

全年地区生产总值突破千亿元，达1100亿元，同比增长15.2%，人均地区生产总值24000元。财政总收入158亿元，增长35%，其中，一般预算收入63亿元，增长27.2%。全社会固定资产投资533亿元，增长30.1%。

工业经济提速增效。牢固确立“工业强则扬州强”的观念，“双创”和“三重”工作深入开展。规模以上工业实现总产值1892亿元，增长31.4%；实现增加值500亿元、销售1806亿元、利税155亿元，分别增长21.3%、32.3%和51%。亏损企业数、亏损总额全面下降。八大产业产值占规模以上工业产值77.8%。规模以上和亿元以上企业数分别比上年增加207个和91个。全年完成工业投入361亿元，增长43.3%。实施亿元以上项目105个，比上年增加44个。高新技术产业实现产值408亿元，增长39.6%。创成省级以上高新技术企业43家、国家级重点新产品和省级高新技术产品149个。新增中国驰名商标1个、中国名牌产品5个、国家免检产品12个。全市建筑企业完成施工总产值550亿元、增加值120亿元，分别增长25.5%和24.8%。荣获“鲁班奖”6个。

农业农村经济平稳增长。大力发展现代高效农业，新农村建设扎实推进。实现农业增加值94亿元，增长5.1%。粮食丰产丰收，总产达245万吨。新增高效农业面积19.3万亩、无公害农产品和绿色食品基地65万亩、“三品”品牌92个。“菜篮子工程”建设取得新成效。扬州鹅、高邮鸭、邵伯鸡获国家级品种认定。农业产业化龙头企业发展势头良好。建成全面小康村120个。新、改建农村公路680多公里，行政村等级公路通达率100%。南水北调三阳河潼河宝应站等一批重点水利工程和灌区改造工程相继建成，战胜了里下河地区严重雨涝灾害。土地复垦开发整理力度加大，实现了耕地总量动态平衡。农业科研、综合开发、粮食流通、农机、气象等工作又有新进展。

服务业加快发展。强化规划引导、政策扶持和机制创新，服务业发展跃上新平台。实现增加值385亿元，增长15.3%。社会消费品零售总额356亿元，增长16%。扬州石化、公铁水、港口等重点物流园区规划通过评审，石化物流园区码头、仓储等项目开工建设。全市港口货物吞吐量5014万吨，增长8.2%。信息服务业实现销售收入41亿元，增长15%。传统特色商贸业持续发展，6家企业获“中华老字号”称号，“扬州师傅”品牌建设得到加强。农村“万村千乡”市场工程进展顺利。全年接待境内外游客1346万人次，实现旅游总收入132亿元，分别增长18.4%和25.9%。房地产业稳定健康发展，全年开发投资83.4亿元，商品房销售面积385万平方米，分别增长14.1%和10.6%。年末金融机构人民币存、贷款余额分别比年初增加133亿元和84亿元。中介、会展和社区服务业等行业发展势头良好。

2、沿江沿河加快开发，对外开放取得成效。

基础设施建设强势推进。沿江高等级公路全面建成通车，扬州二电厂二期扩建工程3号机组并网发电，水电气热等配套设施更加完善。编制了沿河开发总体规划。安大公路三垛以南段贯通，先导段建成通车。各开发园区、乡镇工业集中区建设步伐加快。“八区二园”注册外资实际到帐和外贸出口分别占全市的81.9%和84.2%。新增省级开发区3个。扬州出口加工区顺利通过国家验收，正式封关运行。招商引资力度加大。成功举办2006“烟花三月”国际经贸旅游节，赴欧美、日韩、东南亚和港台、京深沪杭等地开展多种形式的招商推介活动，有效拓展了客商源、信息源、项目源。围绕项目落地和投产达效，加强配套服务、跟踪督查，提高招商项目成功率。全年注册外资实际到帐7.61亿美元，增长44.7%。新批1000万美元以上项目75个。新增民资注册资本215亿元，增长30%。实现外贸出口23.6亿美元，增长23.9%，其中机电产品出口占48%。完成外经营业额1.25亿美元，增长20%。

县域经济实力不断增强。全市县域经济实现地区生产总值767亿元；完成财政收入85.7亿元，增长28.3%。经济结构不断优化，县域三次产业比例由2005年的13.4:56.2:30.4调整为11.7:58.1:30.2。特色产业集聚效应初步显现，已逐步形成石油化工、汽车及零部件、船舶制造、电工电器、服装加工等主导产业，产值占全市规模工业产值的比重达70%。

3、城乡建设步伐加快，人居环境显著改善。

城市规划、建设与管理取得新突破。全面推进城乡规划全覆盖，着手编制扬州市“一体两翼”概念性城市总体规划，完成了广陵新城（河东分区）、东南分区等5个分区规划和城市绿地系统等专项规划，编制了开发区中心片区等控制性详细规划，修编了13个乡镇和76个村庄建设规划。加大城市建设和环境综合整治力度。市区完成城市建设投资72亿元。建成开发路、解放南路、黄金坝北路、三湾大桥等工程，开工建设文昌大桥、渡江南路延伸工程，完成古运河五台山大桥至徐凝门桥段环境整治和景观提升工程。古城保护深入推进，修复卢氏盐商住宅，建成康山文化园一期工程。翻建40条街巷。蜀冈—瘦西湖风景名胜区和新城西区建设步伐加快。新建各类绿地127万平方米。实施天然气置换工程，6万户居民用上天然气。新建垃圾中转场5座。新建、改造公厕18座。新增公交车105辆、公交线路7条。建成数字化城管新平台，首创全国中等城市数字化城管新模式。

节约型社会和生态市建设力度加大。制定出台了《加快发展节约型城市的意见》和《生态市建设行动计划（2006-2010）》。通过国家环保模范城市复查。完成县乡河道疏浚、村庄河塘整治和村庄绿化等年度任务，全市创成3个省级环境优美乡镇。市区完成汤汪污水处理厂二期工程建设，各县（市）都建成了污水处理厂，仪征、江都建成了符合国家标准的垃圾填埋场。市开发区生态工业示范园区规划通过国家级评审。我市被确定为全省循环经济试点市。132家清洁生产试点企业通过审核。开展了集中式饮用水源地整顿专项行动，以及南水北调东线源头、重点化工企业等环境风险排查和整治工作。全市单位地区生产总值能耗下降3.8%，化学需氧量、二氧化硫排放量分别下降3.8%和3.2%。

经过全体市民的共同努力，我市以在古城保护和人居环境改善方面的突出成绩，荣获2006年度中国唯一的“联合国人居奖”。

4、体制改革全面深化，市场经济体制逐步完善。

国有集体企业改革向纵深发展。推进市直工业管理体制改革工作，整合重组金茂化工医药集团、亚星汽车集团、工艺美术集团和工业资产经营管理公司。基本完成市直生产经营型事业单位改革。市政公用事业改革取得进展，完成市燃气总公司合资合作和企业改制。顺利完成联环药业、扬农化工股权分置改革工作。国有资产监管体制不断完善。土地有偿使用制度改革进一步深化。

农村综合改革稳步推进。巩固农村税费改革成果，继续减轻农民负担。贯彻落实“一免三补”等政策，共兑现补贴资金1.56亿元。全市农村合作经济组织进一步发展。深化乡镇农技服务体系改革，逐步完善农经“一站式”窗口服务。加强农村集体财务管理和规范化建设，村务公开继续扩大。

财税、金融和投融资体制等改革不断深化。实施依法治税和社会综合治税，进一步加强税收精细化征管。启动非税收入管理制度改革，继续深化部门预算和国库集中支付改革。制定出台政府投资项目管理和代建制管理办法。成立中小企业担保中心。设立了创业投资引导基金。

5、社会事业全面进步，协调发展水平不断提升。

科技创新能力显著增强。全市组织实施省级以上科技计划项目418项。落实省级以上火炬、星火计划项目103项。宝胜科创、三星电梯等12家企业被认定为国家重点高新技术企业，国家火炬计划扬州汽车及零部件特色产业基地获得科技部批准。科技公共服务平台建设步伐加快，一批信息化项目建成投入使用。与中科院合作共建应用技术研发与产业化中心。与南京大学签订了市校全面合作协议。人才要素有效集聚，全年引进各类人才9500人。全社会知识产权意识明显增强。

教育事业加快发展。全市义务教育阶段入学率保持100%，高中阶段教育毛入学率达87.6%。素质教育进一步推进，教育教学质量不断提高。全部免除农村义务教育阶段学生学杂费，农村办学条件继续改善。新创建9所星级高中、16所省示范初中和省实验小学，扬州中学西区校建成招生。大力发展中高等职业教育，当年招生4.4万人。扬子津科教园区全面投入使用。

文化、卫生、体育等事业持续发展。制定了《扬州文化博览城建设规划纲要》，新增全国重点文物保护单位6处，扬剧等7个项目被列为首批国家级非物质文化遗产。新增“中国工艺美术大师”3名，新评“扬州市工艺美术大师”47名。有线电视入户率达73.5%，有线电视数字化改造工作有序推进。全市疾病预防控制、卫生监督和公共卫生信息网络体系基本建立。城乡社区卫生服务覆盖率分别达78%和40%。新增农村无害化卫生户厕3万户。完成了苏北医院医技楼和市妇幼保健院一期工程建设。全市33个乡镇561个村建成体育健身工程。参加省第十六届运动会，金牌总数和团体总分均获第7名。承办了国际田联竞走挑战赛、鉴真国际马拉松赛等赛事。高邮、仪征、广陵被命名为全省人口计生工作示范市（区）。宝应被评为全国计生优质服务先进县。外事、侨务、台湾事务和统计、档案、方志等工作取得新成绩。我市社会发展总水平列全省第五位。

6、关注民生工作不断加强，人民生活继续改善。

统筹城乡就业取得积极进展。鼓励全民创业，全市新发展私营企业8077家、个体工商户27627户。认真落实就业再就业各项措施，全年采集就业岗位13.1万个，净增就业7.44万人。城镇登记失业率为3.1%。培训下岗失业人员4.7万人、农村劳动力7.9万人，新增转移农村劳动力8.4万人。

社会保障体系逐步完善。全市城镇职工养老保险扩面续保10.8万人，退休人员基本养老金全部实现社会化发放，两次提高企业退休人员养老金发放标准。农村养老保险进一步完善。失业、工伤、生育保险稳步实施。城镇职工医疗保险参保人数达66.4万人。基本解决了破产困难企业7.8万人的医保问题。新型农村合作医疗人口覆盖率达91%。市区“慈济”医院、“慈济”窗口共为7万人次减免医药费610万元。2.7万被征地农民基本生活得到保障。城乡符合低保条件的人员应保尽保，农村五保户集中供养率达47.2%。灾民生活保障、残疾人帮扶工作得到有效落实。新一轮帮扶经济薄弱村和贫困户工作力度进一步加大。

人民生活水平进一步提高。城市居民人均可支配收入12945元，农民人均纯收入5813元，分别增长13.8%和11.5%。居民消费价格总水平基本稳定。城郊又有6个乡镇、30万居民享受区域集中供水。市区建成30万平方米限价商品房，262户低收入家庭领到购房补贴，多层次住房供应体系逐步健全。

7、精神文明和民主法制建设进一步加强，公共服务型政府建设不断深入。

精神文明建设取得新成效。深入贯彻《公民道德建设实施纲要》，积极推进“城市文明建设行动计划”，广泛开展“八荣八耻”教育、“百万市民学礼仪”和窗口行业、服务部门共铸诚信等活动，评选了百名好市民和十佳新人新事，市民素质和社会文明程度进一步提高。未成年人思想道德建设和关心下一代、老龄工作取得新进展。双拥、国防教育和国防后备力量建设工作得到加强，国防（军事）园区启动建设。

民主法制建设不断深入。依法接受市人大及其常委会的工作监督、法律监督，接受市政协的民主监督，积极听取各民主党派、工商联和社会各界意见。认真落实市人大的决议、决定，办理市人大代表建议294件、市政协提案374件，解决落实率进一步提高。加强社区建设和村民自治。实施“五五”普法，广泛开展法制宣传教育。逐步健全突发公共事件应急处理机制。强化社会治安综合治理，“平安扬州”、“法治扬州”建设深入推进。重视抓好安全生产工作，相关控制指标全面下降。认真做好人民群众来信来访工作，积极化解人民内部矛盾。民族、宗教工作取得新进步。

政府自身建设进一步加强。坚持依法行政，不断完善行政执法责任制。继续深化行政审批制度改革，市直部门的行政许可职能归并工作取得明显进展，市级公布保留的行政许可事项实行集中办理。出台了《政府信息公开暂行办法》，开展了行政权力公开透明运行试点。政府公开电话和“寄语市长”回复率、办结率稳步提高。市区为民办实事事项基本完成。认真落实党风廉政建设责任制，廉政建设和反腐败工作取得新成效。

各位代表，这些成绩的取得，是全市人民在中共扬州市委的正确领导下，齐心协力、共同奋斗的结果，是市人大、市政协有效监督和大力支持的结果。在此，我代表市人民政府，向全市人民，向各位人大代表、政协委员，向所有参与、关心和支持扬州建设与发展的同志们、朋友们，表示崇高的敬意和衷心的感谢！

在肯定成绩的同时，我们也清醒地看到，当前经济社会发展中还存在一些值得重视的问题，工作中还有许多薄弱环节，主要是：工业重点产业集聚度不高，自主创新能力不强，具有支撑和带动作用的大企业、大项目不多；统筹区域和城乡发展的任务还很艰巨，农民持续增收的难度加大；经济社会发展与资源环境的矛盾进一步显现；社会生活中还有一些突出的矛盾和问题，影响社会稳定的因素依然存在；政府的服务意识、创新能力和执行能力还需要进一步加强。这些问题都需要我们高度重视，在今后的工作中采取切实有效的措施，认真加以解决。

2007年政府工作任务

今年是党的十七大召开之年，是积极推进社会主义和谐社会建设的重要一年，也是“全面达小康，建设新扬州”的关键一年。政府工作的总体要求是：以邓小平理论和“三个代表”重要思想为指导，以科学发展观为统领，突出又好又快发展主题，着力调整经济结构和转变增长方式，着力推进改革开放和创业创新，着力加强资源节约和环境保护，着力坚持以人为本和解决民生问题，进一步提升产业发展水平，进一步增强县域经济实力，进一步改善人民群众生活，进一步强化政府自身建设，加快建设富裕、文明、秀美、和谐、法治的新扬州。

全市经济和社会发展的预期目标为：地区生产总值增长14%，其中一产增长5%，二产增长15%，三产增长14.5%。财政总收入增长18%，一般预算收入增长18%。全社会固定资产投资增长20%。注册外资实际到帐增长30%，新增民资注册资本增长30%。外贸出口增长15%。社会消费品零售总额增长15.5%。居民消费价格涨幅控制在3%左右。城市居民人均可支配收入增长10%，农民人均纯收入增长9%。城镇登记失业率控制在4%以内。单位地区生产总值能耗下降4%左右。化学需氧量、二氧化硫排放量分别下降3.7%和3.1%。为实现上述预期目标，主要抓好十项工作：

1、进一步做大做强工业经济。

深入推进“双创”、“三重”工作。组织开展“产业发展推进年”活动，以培育大企业、狠抓大项目为重点，着力推动结构优化升级，做大做强石油化工、汽车及零部件、电工电缆等八大主导产业，加快发展半导体照明、太阳能光伏和电子信息等新兴产业。大力实施“双百亿”工程，18个特色产业集群总产值超过800亿元，17家重点企业产值占规模以上工业企业总产值26%。新增产值过50亿元的企业2家以上。

集中力量抓好一批大项目。落实促进工业发展的各项政策，推动国资、外资、民资加大对工业的投入，全年工业投资500亿元。抓好亿元以上项目200个，其中在建项目160个，竣工项目70个以上。重点实施顺大多晶硅、大洋造船等投资超10亿元的项目10个，实友化工、亚东水泥等投资超5亿元的项目20个。

深化企业改革。继续抓好国有集体企业产权制度改革，完善法人治理结构。做好市直工业管理体制改革的后续工作，确保新组建的集团企业化经营、市场化运作。深化企业内部劳动、人事和分配制度改革，转换企业经营机制，增强企业发展活力和竞争优势。加大企业股份制改造和上市扶持力度，力争长青农化、诚德钢管等企业股票上市，扬农、宝胜等上市公司实现再融资。

加强质量管理和品牌建设。鼓励企业采用先进质量标准，大力推进企业和产品质量认证。引导企业争创名牌，鼓励企业在招商引资和项目建设中引进名牌。扩大名牌产品市场规模，提高名牌产品的贡献率。全年新创中国驰名商标1个、中国名牌产品2个。

发展壮大建筑业。进一步深化建筑企业改革改制工作，鼓励优势企业兼并重组，培育壮大江苏华建、邗建集团和扬建公司等重点企业，以及一批地方骨干企业；加大外部市场开拓力度，实施多领域开发、多元化经营。全年建筑企业施工产值、增加值均增长18%以上。

2、扎实推进社会主义新农村建设。

加快发展现代农业。增加对“三农”的投入，加大以工促农、以城带乡、以企带村的力度。继续落实支农惠农各项政策，稳定粮食生产，培育壮大农业优势产业、特色产品。突出抓好设施栽培和规模种养，全年新增高效农业面积30万亩，畜禽规模养殖比重提高5个百分点。培育壮大规模龙头企业，全市销售收入超亿元的龙头企业达30家。抓好“一乡一业、一村一品”，建设100个现代农业产业园区。积极实施农产品出口振兴计划。推进“三品三创”工程，新增无公害农产品基地20万亩、“三品”品牌30个。进一步抓好农业综合开发。加快建设海峡两岸（扬州）农业合作试验区。健全农村社会化服务体系，提高农业信息、技术推广、农业机械、动植物病虫害防控等服务水平。通过土地开发整理，新增耕地1.8万亩，实现占补平衡。

进一步拓宽农民增收渠道。挖掘镇村特色和资源优势，发展劳动密集型工业和家庭手工业。优化乡镇工业集中区布局，集聚发展优势产业。全年乡镇工业总产值、营业收入均增长26%以上。加强农产品市场体系建设，培育运销专业大户、农民经纪人队伍，搞活农产品流通。大力发展农民专业合作经济组织，稳步推行农村社区股份合作，有序推进农村土地股份合作。认真组织农民劳动技能和转移培训，全年培训劳动力5.5万人，新转移劳动力6.5万人。

继续实施农村新五件实事。全年新、改建农村公路600公里、桥梁30座。抓好大型灌区改造、中小型水库除险加固、归江河道水利血防工程和区域水利工程。开展“千村整治、百村示范”活动，抓好县乡河道疏浚和村庄河塘整治。农村绿化造林10万亩。完成农村改厕4万户，新建房无害化卫生厕所配套率达95%以上。建设户用沼气池8000个。规划引导村民集中居住，打造新型农村社区（居民点）。继续深入开展全面小康村建设。

3、大力发展现代服务业。

积极推动生产服务业发展。抓好开发区港口物流、仪征石化物流、江都港口物流、公铁水物流、市区商贸物流等重点物流园区（中心）的建设，积极引进国内外知名的物流企业，培育一批现代物流企业。做好石化物流区申报国家保税物流中心工作。围绕重点市场集聚区建设，发展物流配送。研究制定《关于加快软件与信息服务业发展的意见》。重点发展数字创意、数据加工、呼叫中心等信息服务业。培育发展以工艺品鉴赏、展会、交易和玩具礼品设计等为主体的文化创意产业。进一步优化金融服务，创造良好的金融生态环境，吸引更多的商业银行来扬设立分支机构，协助做好招商银行扬州分行的设立工作。积极发展咨询、评估等中介服务业。

提升旅游业发展水平。加快旅游项目的建设，重点抓好蜀冈—瘦西湖风景名胜区、南河下盐商文化体验区和县域生态旅游区等一批特色旅游项目建设。加快推进古运河城区段综合整治、景观提升和项目开发，丰富古运河水上游览线内涵，推动观光旅游向度假、休闲、体验旅游升级。推进宁镇扬区域旅游协作，以及与长三角、长江沿岸中心城市的旅游合作互动。加强行业管理，提升旅游服务水平。全年接待境内外游客1550万人次，实现旅游总收入155亿元。

做优做强商贸业。进一步提升文昌商圈等商业集中区的经营业态和服务水平，扶持引导重点专业市场加快建设，并带动旅游、餐饮、休闲、娱乐、资讯等相关产业的发展。进一步丰富和创新扬州“三把刀”的文化内涵，鼓励扶持扬州“老字号”做响品牌、做大影响。深化“扬州师傅”品牌创建活动，支持掌握传统技艺的人才创业带徒。进一步抓好“万村千乡”市场工程建设，繁荣农村消费市场。

4、着力壮大县域经济实力。

强势推进强县强镇三年倍增计划。深化完善县域经济社会发展规划，加快改善基础设施条件，全面提升县域经济发展水平、生态人居环境和社会文明程度，实现沿江沿河地区的互动开发、共同发展。今后三年，县域地区生产总值（现价）、全部工业总产值（现价）、财政总收入分别年均增长20%、30%和26%，每年新增2个全国千强镇。力争经过三年的努力，邗江、仪征、江都进入全国百强县（市）行列，高邮、宝应分别进入前180名和200名。

努力提高产业发展水平。依托特色产业、龙头企业，加快发展县域的园区经济、板块经济，促进产业整合，进一步优化生产力布局。加快培育县域工业规模企业群，工业增加值占地区生产总值的比重，高邮、宝应提高2个百分点以上，江都、仪征、邗江提高1个百分点以上。着力培育里下河地区、沿江地区、丘陵地区和城市郊区四大农业板块，推进特色农产品向优势产区集中。充分利用长江、京杭运河和铁路、高速公路的便利，大力发展现代服务业。

积极支持县域经济发展。着力推进城镇化进程，加快县城镇、城镇组团和重点中心镇的建设步伐。实施高速公路联网工程、干线公路畅通工程和农村公路富民工程，加强城镇基础设施、公用设施建设，提高城镇的综合承载能力。进一步落实发展县域经济的政策措施，加大各级财政扶持力度，切实简政放权，为县域经济的发展提供更加优良的服务。

5、努力加快开发开放步伐。

推动招商引资新跨越。坚持量质并举，积极引导境内外资本投向先进制造业、现代服务业、现代农业和高新技术产业，参与现有企业重组。认真举办2007“烟花三月”国际经贸旅游节等节庆活动。瞄准世界500强、跨国公司和国际行业龙头企业，突出产业链招商，强化专业招商、驻点招商、展会招商，争取在重大项目的招商引资上取得新突破。完善考核机制，加大项目跟踪督查力度，提高项目成功率。力争全年注册外资实际到帐达10亿美元。

强化园区建设。加强园区水电气热等设施建设，抓好江都、仪征及化工园区等省级开发区污水处理厂建设，提高园区生产生活配套功能。坚持集约开发，鼓励进园项目节约用地，增加投资强度，提高产出效益；鼓励园区建设标准厂房，吸引中小项目进区发展。创新园区管理机制，促进园区持续、有序发展。“八区二园”注册外资实际到帐均增长40%以上，新开工项目数占全市的80%以上。

推动外贸外经持续增长。优化出口商品结构，提高深加工出口产品比重。支持企业争创出口名牌，提升对外贸易质量和效益。鼓励企业参加境内外商品展销活动，拓宽外销渠道。增加资源性产品和技术设备类产品进口，提升企业技术装备水平。培育和发展外经重点产业和重点企业，规范外经中介机构管理，积极扩大工程承包和劳务合作。进一步发挥海关、检验检疫、海事、边防等口岸查验机构的协同作用，加快“大通关”建设。

6、不断提高民营经济发展水平。

深入实施全民创业工程。进一步落实全民创业的各项政策措施，鼓励千家万户自主创业，支持科技人员、管理人员带头创业，引导现有民营企业二次创业，吸引在外能人回乡创业，引进域外民资投资创业，促进民营经济总量再扩张。全年新发展个体工商户25000户、私营企业8000家。力争新增民资注册资本超过300亿元。

做优做强民营企业。支持民营企业优化产品结构、技术结构、制度结构和人才结构，努力促进重点企业做大做强。引导民营企业走专精特新的发展路子，培育一批中小企业集群。鼓励民营企业扩大经营领域，参与农业产业化、现代服务业和公用事业、社会事业等领域的发展。年产值过亿元的民营企业达200家。

着力优化民营经济发展环境。依托各类开发区（园区）、乡镇工业集中区，建设民营企业发展小区和创业中心，积极打造发展民营经济的新平台。建立健全中小企业信息咨询、人才培训、技术支持等社会化服务体系，帮助民营企业解决生产经营中的困难和问题。加强银企对接，进一步发挥中小企业信用担保中心作用，建立市场化运作的民营企业担保体系，为民营企业和民众创业做好融资服务。

7、切实转变经济增长方式。

加强自主创新。制定和实施科技创新攻关计划，重点抓好一批省级以上重大科技项目的实施，开发一批拥有自主知识产权的新产品。全年新培育省级以上高新技术产品120个、高新技术企业40家，实现高新技术产业产值500亿元。巩固和提升现有国家级扬州高新技术创业服务中心、企业博士后科研工作站、扬州留学生创业园等创新创业平台，建设好中科院扬州应用技术研发与产业化中心和40个企业工程（技术）中心。继续加强与南京大学、扬州大学等高校和驻扬科研机构的合作，积极推进产学研联合，大力引进和培养创新型人才。发挥科技成果转化资金和创业投资引导基金作用，集聚社会资本，支持高技术、高成长企业加快发展。

切实抓好生态市建设和环境保护。实施扬州生态市建设行动计划，积极申报国家生态园林城市，江都、仪征力争创成“国家环保模范城”。加强农村生态环境保护，大力开展环境优美乡镇、生态村、绿色社区的创建，力争创成省级以上环境优美乡镇10个。深化中德生态合作项目建设。继续深入开展整治违法排污企业、保障群众健康环保专项行动，重点抓好188家化工企业专项治理。对159个集中式饮用水源地落实水质保护措施。加强排污总量控制、环境质量监控。加大南水北调东线治污工程建设的督查力度，完成淮河流域23项水污染防治工程。

重视做好资源节约工作。组织全社会参与节约型城市建设。突出抓好重点行业和重点企业的节能减排工作。开展清洁生产先进企业、环境友好企业创建活动。鼓励企业淘汰落后设备与工艺，严格限制高耗能、高耗水、高污染产业。大力发展循环经济，抓好市开发区、化工园区、宝应湖有机农业开发园区等一批循环经济示范园区的建设，继续做好循环经济企业试点工作。积极推广秸秆全量还田、秸秆制气等应用技术。实行最严格的耕地保护制度，提高土地节约集约利用水平。

8、继续加强城乡规划、建设和管理。

积极发挥规划的引导作用。完成扬州市“一体两翼”概念性城市总体规划研究论证，编制主城区控制性详细规划、城市综合交通等专项规划和市域镇村布局规划，合理布局城镇发展、产业用地和生态保护空间，完善城乡基础设施和公共设施配套。理顺城乡规划管理体制，加强规划的执行与管理。

加快重大基础设施建设。开工建设宁通高速六合至江都段和江海高速。抓好扬州港5号码头立项工作，力争年内开工建设。积极做好苏中机场、淮扬镇铁路、五峰山过江通道等重大项目的争取工作。推进国省干线公路建设，安大公路三垛以南段建成通车，三垛以北段开工建设；续建扬天公路仪征段、336省道江都段。继续实施京杭运河“三改二”工程和南水北调工程建设。加大电源、电网项目争取和建设的力度。

进一步完善城市功能。推进新城西区、广陵新城和沿江地区的规划建设，不断拓展城市发展空间。抓好市区市政基础设施和公用设施建设，实施运河北路延伸、运河南路和邗江路改造、解放北路拓宽、扬菱路北段完善等工程，建成文昌大桥。重点解决淮海路南段、南河下、北河下、文汇路西段等路段积水问题，整治新城河等河道，启动乌塔沟区域防洪工程。新增城市绿地100万平方米。规划建设第五水厂。建设新城河路等4座污水提升泵站，配套建设污水管网。市区完成天然气置换工程。

抓好古城保护与利用。实施“双东”街区的改造与复兴工程，建设教场商贸民居民俗文化核心区。整修汪鲁门故居等文保单位，建设北门遗址公园和南门遗址广场。打造“运河名城”，建成康山文化园二期、大王庙广场等7项工程。加大蜀冈—瘦西湖风景名胜区的规划、保护、建设力度，万花园等项目建成并对外开放。

提升城市综合管理水平。进一步发挥数字化城管平台的作用，不断扩大数字化城管范围，提高处置效率。深入开展城管创优活动，强化对环境卫生、违法建设、流动摊点和交通秩序等突出问题的整治。进一步落实属地管理的责任，建立健全长效管理机制。

9、全面促进精神文明建设和社会事业发展。

推进精神文明建设，不断提高公民素质。加强公民思想道德建设和社会主义荣辱观教育，继续深入开展“百万市民学礼仪”等创建活动。做好关心下一代和老龄工作。建设未成年人综合社会实践基地。支持发展志愿服务事业。实施《全民科学素质行动计划纲要》，加强城乡科普宣传，倡导科学、健康、文明的生活方式。重视国防后备力量建设，做好双拥和国防教育工作，争创“全国双拥模范城”五连冠。提高教育质量，办人民满意的教育。贯彻实施《义务教育法》，高水平、高质量普及九年义务教育。推进教育公平，促进教育均衡发展，高中阶段教育毛入学率达88.5%。全面实施义务教育阶段免收学杂费政策。进一步完善扶贫助学机制，确保低收入家庭子女不因贫失学。加大职业技术教育资源整合力度，做强做优职教品牌，打造汽车制造、电子、建筑、烹饪等十大职教重点专业群，建设以扬子津科教园为主的人才培训、实训基地。重视办好特殊教育。

建设“文化扬州”，繁荣文化等事业。推进文化博览城建设，鼓励社会力量参与投资，兴办具有地方特色的陈列馆、纪念馆。保护、开发非物质文化遗产资源，推进瘦西湖及扬州历史城区申遗工作。抓好文艺精品创作，加强基层文化设施建设和文化市场管理，推进农家书香工程和文化信息资源共享工程，丰富群众文化生活。办好全民健身体育节，加快实施行政村体育健身工程。承办好亚洲乒乓球锦标赛、鉴真国际马拉松赛、全国跆拳道冠军赛等。发展广播电视事业和产业，有线电视入户率达78%，加快有线数字电视整体转换步伐。重视做好民族、宗教工作。加强社科研究。切实抓好新闻出版、档案、方志等工作。

完善公共卫生服务体系，提高群众健康水平。进一步健全疾病预防控制、卫生监督、城乡医疗救治和卫生信息网络等体系，加强传染病防治和计划免疫工作。深入开展卫生镇（村）创建活动，农村健康教育普及率达90%以上，新型农村合作医疗覆盖率达92%。大力发展城乡社区新型卫生服务体系，城乡标准化社区卫生服务站达到500个。认真落实“单病种”限价收费等政策措施，切实减轻群众医疗负担。建成苏北医院病房楼和市妇幼保健院急诊、医技楼，完成市急救中心、传染病医院和南方协和医院一期工程建设。稳定低生育水平，抓好农村和流动人口计划生育工作，提高出生人口素质。

10、切实做好关注民生和社会管理工作。

抓好就业再就业工作。落实就业再就业各项政策，加大财政扶持力度，完善城乡一体化的公共就业服务体系。全面推行劳动合同、集体合同制度，加大劳动保障监察力度，切实维护城乡劳动者的合法权益。全年采集就业岗位8.5万个，城镇新增就业4万人，其中下岗失业人员实现再就业1.55万人。培训技术工人3万人。

完善社会保障体系。积极推进“成建制、全覆盖”社会保险扩面工作，全年扩面续保8万人，其中养老保险净增缴费人数3.5万人。完善医疗保险制度，启动实施城镇职工退休人员补充医疗保险，对非职工市区居民通过个人缴费、政府资助的办法实行大病医疗保障。加快推进进城务工农民参加工伤和大病医疗保险工作。适当提高生育保险标准。实施新型农保制度。进一步完善被征地农民基本生活保障工作。切实管好用好社会保险基金，建设一体化的劳动保障综合服务中心。

着力解决弱势群体困难。完善城乡低保制度，建立健全低保标准自然增长机制。农村五保户确保应保尽保，集中供养率60%。深入开展再就业援助工作，力争零就业家庭每户至少有一人就业，对零就业家庭高校毕业生实行保底就业。努力提高医疗救助水平，市区进一步完善低保对象和特困职工医疗救助办法，办好“慈济”医院。建立农村医疗救助基金。切实解决城镇居民的住房困难，继续向符合条件的低收入家庭发放购房补贴或租金补贴。切实做好农村扶贫建房工作。重视发展慈善事业，做好残疾人工作。

继续为民办好实事。抓好市区18件为民办实事项目。翻建、改造街巷30条；新建、改造农贸市场8个；整治老住宅小区4个；新辟、调整公交线路10条，新增公交车100辆；新、改建公厕17座；新建、改造垃圾中转场8座，城郊12个乡镇实行垃圾集中清运处理；完成市区周边乡镇区域集中供水工作等。

切实维护社会稳定。深入开展“平安扬州”、“法治扬州”建设。完善城市治安防控体系，全面落实社会治安综合治理各项措施，不断提高治安防范水平，依法严厉打击各种刑事犯罪。完善“大调解”机制，积极预防和钝化社会矛盾。加强法律援助工作。进一步推进“五五”普法，增强全社会的法制意识。建设高效统一的应急指挥体系，不断增强预防和处置突发公共事件的能力。扎实抓好安全生产工作，全面落实安全生产责任制，坚决防止重特大事故发生。切实加强食品、药品安全及公共场所卫生监督管理工作。进一步做好人民群众来信来访工作，重视解决人民群众合理诉求，有效化解人民内部矛盾。

进一步推进公共服务型政府建设

优质高效的政府服务是又好又快发展的保证。今年政府自身建设将以转变职能为核心，以提升执行力、公信力为目标，以群众满意为标准，着力增强政府机关的服务意识，创新服务方式，提高服务本领。

1、加快转变政府职能。

深化行政管理体制改革。积极运用行政规划、行政指导和行政合同等手段，履行社会管理和公共服务职能。进一步完善公共财政体制，把支出重点更多地放到社会公共需求上来。深入推进财税金融体制及政府投资方式改革。全面推行非税收入管理制度改革。对政府投资项目实行代建制，对社会事业和公益性项目积极尝试政府购买服务，不断提高财政资金的使用效益。全面完成生产经营型事业单位转企改制工作。稳妥推进市属社会公益类事业单位人事、分配制度改革。积极进行乡镇机构改革试点。

加大政务公开力度。扩大公开范围，规范公开内容，拓展公开形式，提高政府工作的透明度。组织实施“阳光工程——规范事权行动”。抓好公用事业单位办事公开工作。进一步完善政府信息公开制度和网上政务公开内容。畅通政府与群众沟通交流渠道，提高政府公开电话和“寄语市长”的回复率和办结率。制定出台《政务公开责任追究办法》，确保行政权力公开公正运行。

2、着力提高政府执行力。

狠抓工作落实。进一步加强学习，解放思想，增强大局意识、责任意识和创新意识，提高开拓创新能力、协调服务能力和执行落实能力。进一步加强团队建设，发挥团队作用，增强团队功能；部门领导要以身作则，以高尚的人格、务实的工作、清廉的作风去影响和带领好团队；部门之间要加强协调配合，形成整体合力，确保政令畅通。进一步强化目标管理、效能监察和责任追究，制定出台《行政首长问责暂行办法》，完善部门工作责任制、督查制度和奖惩激励机制，加大工作推进力度。

提高服务效率。强化首问负责制、服务承诺制、岗位责任制、限时办结制，加快推进网上审批，全面实施与“两集中、两到位”相配套的改革措施，试行并推广联合查勘制、重大事项跟踪帮办制，进一步方便群众、方便投资者。深入开展“走门串户、联系企业、联系群众”等活动，围绕企业、投资者和广大市民群众关心的重点、难点、热点问题，强化协调服务，提高服务水平。

3、切实增强政府公信力。

坚持科学民主决策。进一步完善政府决策规则，建立公众参与、专家论证和政府决定相结合的决策机制，对涉及经济社会发展全局的重大事项，广泛征询意见，充分讨论协商；对专业性、技术性较强的重大事项，认真进行专家论证、技术咨询、决策评估；对同群众利益密切相关的重大事项，实行公示、听证等制度，扩大人民群众的参与度。完善决策监督机制，建立健全行政决策责任追究制度，实现决策权与决策责任相统一，做到政府的各项决策和对人民的承诺言出必行、行必见效。

坚持依法行政。认真贯彻《监督法》，自觉接受人大及其常委会的工作监督和法律监督，支持人民政协履行职能。加强与人大代表和政协委员的联系，主动听取民主党派、工商联、无党派人士和各人民团体的意见，真正把各项公务活动置于人民的监督之下。完善行政执法运行机制，基本建成权责明确、行为规范、监督有效、保障有力的行政执法体制。深入推行行政执法责任制，纠正少数公务人员行政不作为、乱作为行为，做到严格执法、文明执法、公正执法。

坚持廉洁从政。认真学习贯彻胡锦涛总书记在中纪委第七次全体会议上讲话精神，切实加强政府廉政建设，坚决纠正损害群众利益的不正之风，扎实开展治理商业贿赂专项工作，建立健全从源头上防范腐败的体制机制，更好地用法律和制度规范政府工作人员的从政行为。加强干部经济责任审计和行政监察工作。遵守廉洁自律各项规定，崇尚艰苦奋斗、勤俭节约的作风，坚决反对各种奢侈浪费和不廉洁行为，努力建设一支政治坚定、业务精通、清正廉洁、作风优良的公务员队伍。

各位代表，今年是本届政府任期的最后一年，全面完成本届政府的目标任务，向人民群众交一份满意的答卷，任务重、责任大。让我们在中共扬州市委的领导下，高举邓小平理论和“三个代表”重要思想伟大旗帜，贯彻落实科学发展观，加快构建社会主义和谐社会，以更加振奋的精神，更加昂扬的斗志，开拓创新，扎实工作，为“全面达小康，建设新扬州”而努力奋斗！
